# Genetic and Serological Survey of Sarcoptic Mange (*Sarcoptes scabiei*) in Wild Boars (*Sus scrofa*) in South Korea

**DOI:** 10.3390/ani14233490

**Published:** 2024-12-03

**Authors:** Sanghyun Lee, Garam Kim, So-Jeong Kim, Weon-Hwa Jheong, Dong-Hyuk Jeong

**Affiliations:** 1 National Institute of Wildlife Disease Control and Prevention (NIWDC), Gwangju 62407, Republic of Korea; ishmael6410@korea.kr (S.L.); garam1204@korea.kr (G.K.); th3555@korea.kr (S.-J.K.); purify@korea.kr (W.-H.J.); 2 Laboratory of Wildlife and Conservation Medicine, College of Veterinary Medicine, Chungbuk National University, Cheongju 28644, Republic of Korea; 3 Chungbuk Wildlife Center, Cheongju 28116, Republic of Korea

**Keywords:** *Sarcoptes scabiei*, sarcoptic mange, wild boar, seroprevalence, South Korea

## Abstract

Sarcoptic mange, caused by *Sarcoptes scabiei*, has been reported in various wildlife species, with the potential to impact populations. While it has been documented in some wildlife species and domestic animals, it has not been previously reported in wild boars in South Korea. In March and April 2022, skin samples from nine wild boars and one raccoon dog with suspected sarcoptic mange were submitted to the NIWDC. These samples were tested and confirmed the presence of *S. scabiei* in six wild boars and one raccoon dog. Genetic analysis of the *cox-1* gene from mites indicated that the gene was identical and belonged to the same haplotype in both host species. Serological survey of 658 wild boars, collected from March 2022 to February 2023, revealed a nationwide seroprevalence of 5.47%. The highest seroprevalence was in Gyeongsangnam-do (9.82%); however, regional differences in seroprevalence across the country were not statistically significant, suggesting the disease may be endemic across South Korea. Seroprevalence was highest in spring (16.84%), likely due to increased contact during winter. This study provides fundamental data on the genetic characteristics and distribution of *S. scabiei* mites in South Korean wild boars, emphasizing the need for continued surveillance to prevent further spread.

## Simple Summary

Sarcoptic mange is a highly contagious disease affecting various animals. In this study, skin samples from wild boars in South Korea confirmed the presence of *Sarcoptes scabiei*, the mite that causes sarcoptic mange, and genetic analysis of the *cox-1* gene showed that the mites shared an identical haplotype. A serological survey of 658 wild boars across South Korea found that 5.47% of the animals had antibodies to *Sarcoptes scabiei*, indicating prior exposure. These findings indicate that sarcoptic mange may be prevalent within the wild boar population in South Korea, highlighting the importance of ongoing monitoring and control efforts to protect wildlife.

## 1. Introduction

Sarcoptic mange, caused by *Sarcoptes scabiei* and known as ‘scabies’ in humans, is a highly contagious skin disease in mammals. This disease has been recognized as a neglected tropical disease in human health, highlighting its significance [1]. There have been increasing reports of sarcoptic mange in a growing number of wildlife species, and it is now considered a new threat to wildlife populations [2,3]. Sarcoptic mange is characterized by intense pruritus, erythematous rashes, papule formation, seborrhea, and alopecia [4]. These symptoms can affect an infested animal’s ability to regulate body temperature and energy metabolism, making them more susceptible to bacterial infections and potentially leading to death, which has contributed to significant population declines in many wildlife species [3,5,6,7]. Although *Sarcoptes scabiei* is classified as a single species taxonomically and is divided into several variants due to its host specificity [8], multiple cases of cross-transmission between different host species have been reported [9,10,11,12], raising concerns regarding the potential for cross-species transmission. Notably, cross-species transmission of *Sarcoptes scabiei* has been reported in 42% of species within the Artiodactyla order, including interspecies transmission between Iberian ibex and wild boar, suggesting that such transmission may occur more frequently within this group than in others [3,9]. However, research on sarcoptic mange in wild boars remains relatively limited, highlighting the need for further study in this species [13,14].

In South Korea, cases of sarcoptic mange have been reported in free-ranging wild animals such as raccoon dogs (*Nyctereutes procyonoides*), long-tailed gorals (*Naemorhedus caudatus*), and red foxes (*Vulpes vulpes*) [15,16,17], as well as in livestock or companion animals such as domestic pigs, dogs, and a pet rabbit [18,19,20]. Although multiple cases of sarcoptic mange in wild boars have been reported in Europe and East Asia [4,8,21,22,23], there have been no reported cases of sarcoptic mange in wild boars in South Korea. Moreover, wild boars are widely distributed across the world [24] and inhabit various environments, ranging from forests to urbanized areas in South Korea [25,26]. It is thus hypothesized that wild boars may be susceptible to *Sarcoptes scabiei* infestations, with anecdotal reports suggesting that such infestations have occurred at least since the mid-2010s in South Korea.

This study reports the first confirmed case of sarcoptic mange in wild boars in South Korea. A genetic analysis was conducted using the mitochondrial *cox-1* gene derived from *Sarcoptes scabiei* in wild boars, comparing it with sequences from *Sarcoptes scabiei* mites found in various host species in South Korea and other countries. Furthermore, the seroprevalence was assessed using serum samples from wild boars in South Korea. This study aims to enhance our understanding of the epidemiology of sarcoptic mange in wildlife and provide fundamental information for its management.

## 2. Materials and Methods

### 2.1. Sample Collection

From March to April 2022, nine wild boars displaying symptoms suggestive of sarcoptic mange were reported to the National Institute of Wildlife Disease Control and Prevention (NIWDC), located in Gwangju, South Korea. These reports came from the regions of Yeongwol in Gangwon-do, as well as Mungyeong and Cheongsong in Gyeongsangbuk-do (‘-do’ refers to province). During the same period, a raccoon dog exhibiting signs of sarcoptic mange was also submitted to the NIWDC from Goesan in Chungcheongbuk-do. To confirm the presence of *Sarcoptes scabiei* mites and conduct genetic analysis, skin tissue samples were collected from those animals (Table 1). The wild boar samples used in this study were collected by local hunters or the wild boar carcass search teams for African swine fever (ASF) surveillance. After collection, the samples were transferred to the NIWDC and stored at −20 °C.

For serological analysis, 658 wild boar serum samples were randomly selected from the samples collected nationwide for African swine fever surveillance at the NIWDC between March 2022 and February 2023. Piglets weighing less than 20 kg [27,28] were excluded from consideration during the sample selection process. This exclusion was based on the manufacturer’s manual for the commercial ELISA kit, SARCOPTES-ELISA 2001^®^ Pig (AFOSA GmbH, Blankenfelde-Mahlow, Germany), used in this study. The blood samples were collected by local hunters and transported to the NIWDC, where serum was separated and then stored at −70 °C. The selected serum samples were categorized by region as follows: GG: Gyeonggi-do (including Seoul and Incheon, though no samples were actually collected from Incheon); GW: Gangwon-do; CN: Chungcheongnam-do (including Daejeon and Sejong); CB: Chungcheongbuk-do; JB: Jeollabuk-do; JN: Jeollanam-do (including Gwangju); GB: Gyeongsangbuk-do (including Daegu); GN: Gyeongsangnam-do (including Busan and Ulsan). Additionally, Jeju Island was excluded from the study area (Table 2).

All wild boar samples used in this study tested negative for African swine fever in prior examinations.

### 2.2. Microscopic Examination and Genetic Analysis

Skin tissue samples from the raccoon dog and wild boars were cut into 2–5 mm sections and reacted in 1000 μL of 10% KOH solution for 30 min. Bead beating (4 m/s, 20 s per run, repeated 3 times) was performed using FastPrep^®^-24 (MP Biomedicals, Santa Ana, CA, USA), followed by centrifugation at 10,000 rpm for 1 min. Then, 30 μL of the supernatant was dispensed onto a microscope slide, covered with a coverslip, and observed under an optical microscope (Eclipse Ni-U, Nikon, Tokyo, Japan). After observing the mites under the microscope, photographs were taken using the digital imaging system (Digital Sight 10, Nikon, Tokyo, Japan).

After removing the supernatant used for microscopic observation of *Sarcoptes scabiei* mites, the remaining pellets were used for genomic DNA extraction using the HiGene™ Genomic DNA Prep Kit (Column type, BIOFACT, Daejeon, South Korea) following the manufacturer’s instructions. PCR was performed to target the *cox-1* gene region of *Sarcoptes scabiei*, using the Applied Biosystems SimpliAmp thermal cycler (Thermo Fisher Scientific, Waltham, MA, USA) (Table 3). PCR products were examined by 1% agarose gel electrophoresis, purified and sequenced by BIOFACT (Daejeon, South Korea) through Sanger sequencing. Species identification was performed via the NCBI BLAST program. After sequencing, nucleotide sequences were compared using the CLUSTAL Omega alignment program [29], and phylogenetic analysis was conducted with MEGA software version X [30]. Evolutionary distances were calculated using the Tamura-Nei model algorithm [31], and Bootstrap analysis was performed with 1000 replicates. Haplotypes were identified through DnaSP v6.12.03 [32].

### 2.3. Serological and Statistical Analysis

Antibody tests were conducted on 658 wild boar serum samples using the commercially developed ELISA kit (SARCOPTES-ELISA 2001^®^ Pig, AFOSA GmbH, Blankenfelde-Mahlow, Germany), according to the manufacturer’s instructions. The kit was originally designed for antibody detection in domestic pigs, with a sensitivity of 94% and specificity of 97%. In wild boar samples, it demonstrated a sensitivity of 75% and a specificity of 80% [13]. The results of the antibody test were grouped by region to measure seroprevalence, and the 95% confidence intervals for the seroprevalence were calculated using the Clopper–Pearson method [33]. The capture locations of positive and negative wild boars were geographically mapped and visualized using QGIS software (ver. 3.34 Prizren). Fisher’s exact test was employed to examine differences in seroprevalence across regions. In addition, the serum samples were grouped by sex, age, and hunting season to examine whether there were differences in seroprevalence among these groups. Age was classified based on body weight: juveniles (20 kg to less than 40 kg), subadults (40 kg to less than 60 kg), and adults (60 kg or more) [28]. The hunting seasons were categorized as follows: spring (March–May), summer (June–August), fall (September–November), and winter (December–February). Pearson’s Chi-Square test was used to analyze differences in the seroprevalence for *Sarcoptes scabiei* in wild boars based on sex, age, and hunting season. To assess the effects of each variable (sex, age, and hunting season) on seroprevalence, a multivariate logistic regression analysis was performed. Forward selection based on the Akaike Information Criterion (AIC) was used to identify the optimal model, starting with the null model and sequentially adding variables. The reference category for the analysis was set as “male” for sex, “juvenile” for age, and “winter” for season. All statistical analyses were performed using R software (version 4.4.1; R Core Team, Vienna, Austria).

## 3. Results

### 3.1. Identification and Phylogenetic Analysis of Sarcoptes scabiei

**Figure 1:**
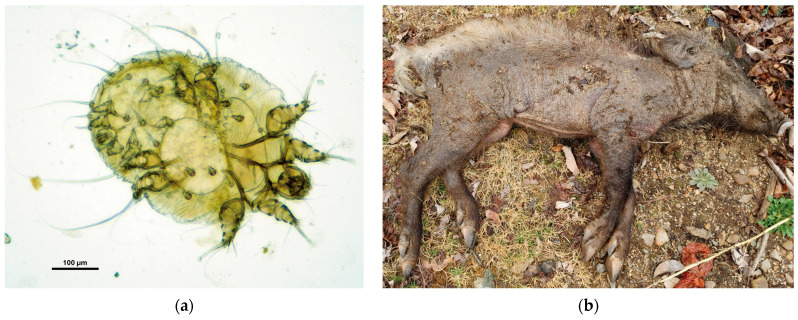
Microscopic images of *Sarcoptes scabiei* mite and carcass of a wild boar suspected of sarcoptic mange. (**a**) The *Sarcoptes scabiei* mite, magnified at 100×, was confirmed from the wild boar carcass (MG01WB). (**b**) The wild boar carcass (MG01WB), suspected of sarcoptic mange, was reported to the NIWDC. This image was submitted alongside the diagnostic request to the NIWDC’s Wild Animal Disease Information System (wadis.go.kr, accessed on 19 March 2022) for ASF testing.

**Figure 2:**
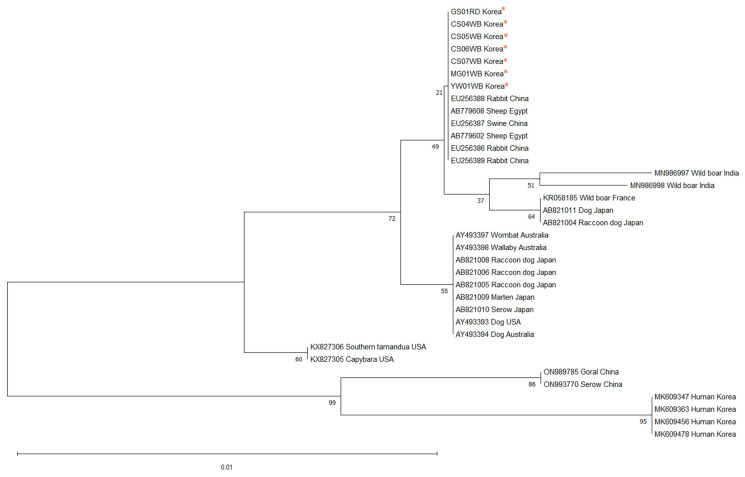
Phylogenetic tree of *Sarcoptes scabiei* from the raccoon dog and wild boars in South Korea, based on mitochondrial *cox-1*. Sequences of *Sarcoptes scabiei* marked by red stars represent the raccoon dog and wild boar samples used in this study. Sequences of *Sarcoptes scabiei* from various host species in different regions, obtained from NCBI GenBank, are included for comparison.

*Sarcoptes scabiei* mites were identified in 7 (GS01RD, CS04WB, CS05WB, CS06WB, CS07WB, MG01WB, YW01WB) out of the 10 samples (Figure 1), and PCR analysis of these skin samples revealed the amplification of the *cox-1* gene of *Sarcoptes scabiei*. Mites were not detected in the remaining three samples (CS01WB, CS02WB, CS03WB) through both microscopic examination and PCR. The genetic analysis showed that the *Sarcoptes scabiei* mites from the raccoon dog and wild boars were identical in the *cox-1* gene region. The phylogenetic analysis of *cox-1* revealed that the *Sarcoptes scabiei* mites from the raccoon dog and wild boars identified in this study were closely related to those from sheep (AB779602, AB779608) in Egypt [34], rabbits (EU256386, EU256388, EU256389), and pigs (EU256387) in China, sharing the same haplotype, while the mites from humans (MK609347, MK609363, MK609456, MK609478) in South Korea were found to be phylogenetically distant, with a different haplotype (Figure 2).

### 3.2. Seroprevalence and Influencing Factors of Sarcoptes scabiei in Wild Boars

**Figure 3:**
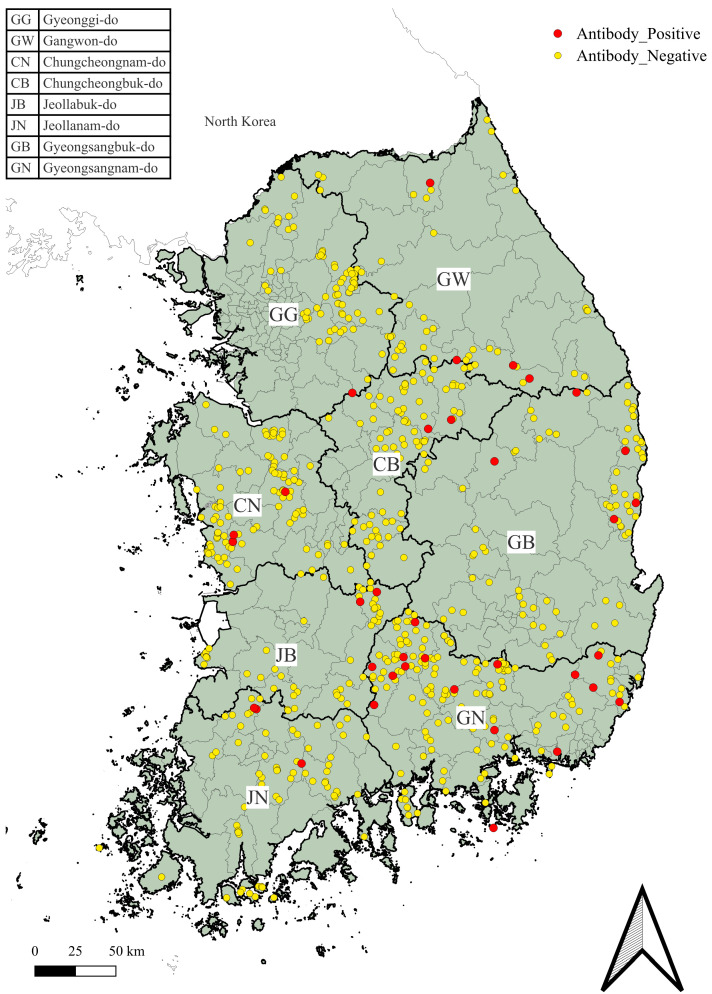
Nationwide map of ELISA results for sarcoptic mange in wild boars. This map displays the results of *Sarcoptes scabiei* ELISA tests across South Korea, indicating where wild boars tested positive or negative for the antibodies.

The antibody test confirmed that 36 out of 658 wild boar serum samples collected nationwide were positive, resulting in a seroprevalence of 5.47% (36/658), with *Sarcoptes scabiei* antibodies detected in wild boars across all survey areas (Figure 3). Regionally, the highest seroprevalence for *Sarcoptes scabiei* in wild boars was observed in the GN region at 9.82% (16/163), while the lowest rate was observed in the GG region at 1.39% (1/72) (Table 4). Fisher’s exact test results (*p* = 0.197) indicated that there was no significant difference in sarcoptic mange seroprevalences across the country.

Females exhibited a slightly higher seroprevalence (5.63%; 17/302) compared to males (5.34%; 19/356), but Pearson’s Chi-Square test results with Yates’ continuity correction (χ^2^ ≈ 0.000, *p* = 1.000) indicated no significant difference between sexes. By age, the seroprevalence was 4.20% (6/143) in juveniles, 4.46% (7/157) in subadults, and higher in adults at 6.42% (23/358); however, Pearson’s Chi-Square test results (χ^2^ = 1.390, *p* = 0.499) showed no significant differences by age. In contrast, the seroprevalence based on the hunting season was highest in spring at 16.84% (16/95) and lowest in winter at 3.17% (6/189), with Pearson’s Chi-Square test results (χ^2^ = 27.903, *p* < 0.01) revealing a highly significant difference depending on the hunting season (Table 5).

In the final model of the multivariable logistic regression analysis using the forward selection approach based on AIC (AIC: 266.71), spring was identified as the most influential factor for seroprevalence (OR: 6.18, 95% CI: 2.44–17.75, *p* < 0.001). This indicates that seroprevalence during spring was significantly higher compared to the reference category, winter. In contrast, summer and fall showed no significant differences compared to winter (*p* = 0.95 and *p* = 0.67, respectively). The final model did not include interaction terms, which suggests that the results are based on the main effects of each variable. These findings align with the results of the univariable analysis based on Pearson’s Chi-Square test (Table 6).

## 4. Discussion

Sarcoptic mange has been documented in raccoon dogs, long-tailed gorals, and red foxes among wildlife, as well as in pigs, dogs, and a pet rabbit among livestock or companion animals in South Korea [15,16,17,18,19,20]. In contrast, while numerous cases have been reported in wild boars across Europe and other countries, no such cases have been recorded in wild boars in South Korea [4,8,21,22,23,28]. Therefore, this study is the first to investigate the genetic characteristics and nationwide distribution of *Sarcoptes scabiei* mites in wild boars in South Korea.

In this study, the genetic analysis of *Sarcoptes scabiei* derived from the raccoon dog and wild boars revealed that the mites from these two species in South Korea share identical sequences in the *cox-1* gene, indicating the same haplotype. Various haplotypes of *cox-1* in *Sarcoptes scabiei* have been reported in South Korean wildlife, and the *cox-1* sequence of *Sarcoptes scabiei* found in the raccoon dog and wild boars in this study is presumed to be identical or similar to the *cox-1* sequences of *Sarcoptes scabiei* reported in long-tailed gorals (Yangyang in Gangwon-do and Uljin in Gyeongsangbuk-do) [16,17], although a direct comparison was not made in this study. The *cox-1* sequence found in the raccoon dog and wild boars in this study appears to be different from the haplotypes commonly observed in South Korea as well as globally [16,17,22,35,36]. These findings align with previous studies suggesting that *Sarcoptes scabiei* infestations in South Korea may not follow species-specific patterns in wildlife, and they highlight the potential for cross-species transmission among wildlife populations in the country [16,17]. Moreover, given that raccoon dogs and wild boars are known to be evenly distributed throughout the Korean Peninsula [26], this suggests that these two species could potentially serve as sources of transmission for *Sarcoptes scabiei* among wildlife in South Korea. However, the analysis of genetic characteristics using only *cox-1* does not provide sufficient evidence to support the possibility of interspecies transmission. Consequently, further investigation utilizing microsatellite analysis is required to elucidate the interspecies transmission dynamics of *Sarcoptes scabiei* derived from diverse wild animal hosts [9,10].

A total of 658 wild boar serum samples, which were submitted to the NIWDC as a part of African swine fever surveillance from March 2022 to February 2023, were tested for sarcoptic mange seroprevalence using a commercial ELISA kit. The seroprevalence for *Sarcoptes scabiei* in wild boars across South Korea was 5.47%, with at least one antibody-positive case confirmed in all surveyed areas. The highest seroprevalence was observed in the GN region at 9.82%, followed by the GW region at 6.82%. In contrast, the lowest seroprevalence was recorded in the GG region at 1.39%. When comparing sarcoptic mange seroprevalences by region using Fisher’s exact test, no statistically significant differences were found across the country. This suggests that sarcoptic mange may be evenly distributed and endemic among wild boars throughout South Korea, regardless of region. Additionally, Pearson’s Chi-Square test and multivariate logistic regression analysis were performed to examine whether sex, age, and hunting season influenced the seroprevalence of *Sarcoptes scabiei* in wild boars by grouping each variable. The results indicated that hunting season had a highly significant effect on seroprevalence, with spring being identified as the most influential season (OR: 6.18, 95% CI: 2.44–17.75, *p* < 0.001). This finding aligns with a previous study [37], which reported higher seroprevalence during spring and summer, suggesting a potential seasonal pattern in the epidemiology of sarcoptic mange in wild boars. However, considering that the ELISA kit can detect sarcoptic mange approximately 5–6 weeks after infestation [38], and antibodies can persist for up to 12 months (according to the ELISA kit manufacturer), this does not necessarily mean that spring is the peak season for wild boar infestations. It has been reported multiple times that sarcoptic mange is influenced by seasonal factors, with severity tending to increase during winter [3,39,40,41]. Direct contact is a key transmission factor for sarcoptic mange in environments unsuitable for the mite (e.g., snow) [3,42], and winter is a major mating season for wild boars in South Korea [27]. Therefore, it can be inferred that increased contact during mating, combined with harsh winter conditions, may contribute to a rise in sarcoptic mange among wild boars in South Korea. Consequently, it can be speculated that the increase in sarcoptic mange during winter leads to higher antibody prevalence rates in the spring, and, therefore, it is necessary to devise management strategies for sarcoptic mange not only for the spring, when the seroprevalence is high, but also for the winter, when infestations are likely to intensify. While this study utilized samples collected over the course of 1 year, continuous monitoring is necessary to fully understand the seasonal effects of sarcoptic mange and to further evaluate potential interaction effects between season and age in shaping seroprevalence patterns.

This study only compared the genetic sequences of *Sarcoptes scabiei* from wild boars and a single raccoon dog, focusing on the *cox-1* gene. Thus, it is important for future studies to analyze the genetics of *Sarcoptes scabiei* from a broader range of wildlife in Korea using more diverse genetic markers, such as microsatellites, to better assess the potential for cross-species infestations and gain a deeper understanding of the genetic diversity of *Sarcoptes scabiei*. Additionally, this study only investigated the seroprevalence for *Sarcoptes scabiei* in wild boars based on sex, age, season, and region. However, further research should include spatiotemporal analysis that considers various factors such as climate, geographical characteristics, and habitat suitability for a more comprehensive understanding. Furthermore, disease–ecological research on wild boars will contribute to more effective wildlife disease management. Through this approach, it will be possible to identify epidemiological links between sarcoptic mange and other diseases affecting wild boar populations, such as African swine fever.

## 5. Conclusions

Several studies in South Korea have recognized the importance of researching sarcoptic mange [15,16,17,18,19,20], and it has been identified as a disease affecting wildlife populations in South Korea [43]. However, no studies have been conducted on *Sarcoptes scabiei* in wild boars. In this study, we analyzed the *cox-1* gene of *Sarcoptes scabiei* in wild boars to identify which haplotypes are present within the population in South Korea. Furthermore, we conducted a nationwide serological survey to assess the seroprevalence of sarcoptic mange, which suggested that the disease may be endemic among wild boars in South Korea. These results suggest that wild boars, which are widely distributed across the country, could potentially act as a source of *Sarcoptes scabiei* transmission.

The management of sarcoptic mange in wildlife is particularly important for endangered species. While sarcoptic mange may not have a long-term impact on species with stable populations, it can be fatal for endangered species or those with small habitats [8]. Since the 2000s, restoration projects for endangered species, such as the Asiatic black bear (*Ursus thibetanus*), long-tailed goral (*Naemorhedus caudatus*), and red fox (*Vulpes vulpes*), have been underway in South Korea [44]. For these species, cases of sarcoptic mange have been reported in South Korea and Japan [16,17,45]. Thus, it is important to assess the infestation status of *Sarcoptes scabiei* in endangered wildlife in areas where wild boar infestations have been confirmed and to strengthen management efforts.

## Figures and Tables

**Table 1:** The raccoon dog and wild boar samples submitted to the NIWDC with suspected symptoms of sarcoptic mange.

ID No.	Type	Species	Region
GS01RD	Carcass	Raccoon dog	Goesan, CB
CS01WB	Captured	Wild boar	Cheongsong, GB
CS02WB	Captured	Wild boar	Cheongsong, GB
CS03WB	Captured	Wild boar	Cheongsong, GB
CS04WB	Captured	Wild boar	Cheongsong, GB
CS05WB	Captured	Wild boar	Cheongsong, GB
CS06WB	Captured	Wild boar	Cheongsong, GB
CS07WB	Captured	Wild boar	Cheongsong, GB
MG01WB	Carcass	Wild boar	Mungyeong, GB
YW01WB	Carcass	Wild boar	Yeongwol, GW

The abbreviations used in this table refer to the following provinces: GW for Gangwon-do, CB for Chungcheongbuk-do, GB for Gyeongsangbuk-do.

**Table 2:** Number of wild boar serum samples by region used for serological analysis.

Region	GG	GW	CN	CB	JB	JN	GB	GN	Total
N. of Samples	72	44	99	84	48	69	79	163	658

The abbreviations used in this table refer to the following provinces: GG for Gyeonggi-do, GW for Gangwon-do, CN for Chungcheongnam-do, CB for Chungcheongbuk-do, JB for Jeollabuk-do, JN for Jeollanam-do, GB for Gyeongsangbuk-do, and GN for Gyeongsangnam-do.

**Table 3:** Target gene region and sequence for PCR.

Target Gene		Sequence (5′ → 3′)	PCR Condition
*cox-1* (518 bp)	F	TGATTTTTTGGACACCCGGAAGT	95 °C for 15 min; 35 cycles of 95 °C for 20 s, 55 °C for 30 s, 72 °C for 30 s; 72 °C for 5 min
	R	GAAGGTTTTAAAAAATAACCTGTAAACATTATGTATCAAAAAGAAAAACC	

**Table 4:** Seroprevalences for *Sarcoptes scabiei* in wild boars by region.

Region	Pos/N	Prev in %	95% CI
GG	1/72	1.39	0.04–7.5
GW	3/44	6.82	1.43–18.66
CN	3/99	3.03	0.63–8.60
CB	3/84	3.57	0.74–10.08
JB	2/48	4.17	0.51–14.25
JN	3/69	4.35	0.91–12.18
GB	5/79	6.33	2.09–14.16
GN	16/163	9.82	5.72–15.45
Total	36/658	5.47	3.86–7.49

The abbreviations used in this table refer to the following provinces: GG for Gyeonggi-do, GW for Gangwon-do, CN for Chungcheongnam-do, CB for Chungcheongbuk-do, JB for Jeollabuk-do, JN for Jeollanam-do, GB for Gyeongsangbuk-do, and GN for Gyeongsangnam-do. ‘Pos’ refers to positive cases for *S. scabiei* antibodies, ‘N’ indicates the number of samples, ‘Prev’ indicates prevalence, and ‘95% CI’ represents the 95% confidence interval calculated using the Clopper–Pearson method.

**Table 5:** Seroprevalences for *Sarcoptes scabiei* in wild boars by sex, age, and hunting season.

Variable	Group	Pos/N	Prev in %	95% CI
Sex	Male	19/356	5.34	3.24–8.21
	Female	17/302	5.63	3.31–8.86
Age	Juvenile	6/143	4.20	1.56–8.91
	Subadult	7/157	4.46	1.81–8.97
	Adult	23/358	6.42	4.12–9.48
Hunting Season	Spring	16/95	16.84	9.94–25.9
	Summer	4/121	3.31	0.91–8.25
	Fall	10/253	3.95	1.91–7.15
	Winter	6/189	3.17	1.17–6.78
Total		36/658	5.47	3.86–7.49

‘Pos’ refers to positive cases for *S. scabiei* antibodies, ‘N’ indicates the number of samples, ‘Prev’ indicates prevalence, and ‘95% CI’ represents the 95% confidence interval calculated using the Clopper–Pearson method.

**Table 6:** Results of the multivariable logistic regression analysis using the forward selection approach based on AIC for seroprevalence of *Sarcoptes scabiei* (Reference category: Winter).

Variable	Estimate	*p*-Value	Odds Ratio (95% Confidence Interval)
Intercept	−3.42	<0.001	-
Season (Spring)	1.82	<0.001	6.18 (2.44–17.75)
Season (Summer)	0.04	0.949	1.04 (0.26–3.73)
Season (Fall)	0.23	0.665	1.26 (0.46–3.75)

## Data Availability

The raw data supporting the conclusions of this article will be made available by the authors on request.
